# The effect of metabolic syndrome on postoperative complications and long-term survival of patients with colorectal cancer

**DOI:** 10.3389/fonc.2023.1036458

**Published:** 2023-06-26

**Authors:** Ce Zhu, Chenchen Mao, Wentao Cai, Jingwei Zheng, Hui Yang, Tao You, Jian Chen, Yaojun Yu, Xian Shen, Liyi Li

**Affiliations:** ^1^ Department of Gastrointestinal Surgery, The Second Affiliated Hospital, Wenzhou Medical University, Wenzhou, Zhejiang, China; ^2^ Department of Microbiology and Immunology, School of Basic Medical Sciences, Institute of Molecular Virology and Immunology, Wenzhou Medical University, Zhejiang, China; ^3^ Department of Gastrointestinal Surgery, The First Affiliated Hospital, Wenzhou Medical University, Wenzhou, Zhejiang, China

**Keywords:** metabolic syndrome, colorectal cancer, overall survival, postoperative complications, propensity score matching

## Abstract

**Background:**

Metabolic syndrome (MetS) is associated with poor prognosis in many cancers. However, the relationship between metabolic syndrome and overall survival (OS) in patients with colorectal cancer (CRC) remains unclear. We aimed to comprehensively analyze whether MetS could affect postoperative complications and long-term survival in patients with CRC.

**Methods:**

We included patients who underwent CRC resection at our center between January 2016 and December 2018. Bias was reduced through propensity score matching analysis. Patients with CRC were divided into the MetS and non-MetS groups based on whether they had MetS. Univariate and multivariate analyses were used to identify risk factors affecting OS.

**Results:**

We included 268 patients; among them, 120 were included for further analysis after propensity score matching. There were no significant between-group differences in the clinicopathological features after matching. Compared with the non-MetS group, the MetS group had a shorter OS (P = 0.027); however, there was no significant between-group difference in postoperative complications. Multivariate analysis revealed that MetS (hazard ratio [HR] = 1.997, P = 0.042), tumor-node-metastasis stage (HR = 2.422, P = 0.003), and intestinal obstruction (HR = 2.761, P = 0.010) were independent risk factors for OS.

**Conclusions:**

MetS affects the long-term survival of patients with CRC without affecting postoperative complications.

## 1 Introduction

Worldwide, colorectal cancer (CRC) is the third most common malignancy and the fourth leading cause of cancer-related deaths (1). In the next decade, the global CRC burden is expected to increase by > 50%, which accounts for millions of new cases and deaths (2).

Metabolic syndrome (MetS) is characterized by several metabolic disorders, including hypertension, abnormal glucose metabolism, obesity, and dyslipidemia (3). Individuals with MetS are more likely to develop numerous diseases, including cardiovascular diseases and nephropathy (4). Socio-economic and lifestyle changes as well as the aging population have led to a sharp increase in the worldwide incidence of MetS (5). Patients with CRC have an increased risk of MetS (6, 7). Studies on the relationship between MetS and outcomes after CRC surgery (8, 9) have mainly focused on short-term prognosis, with the relationship between MetS and long-term survival after CRC surgery remaining unclear. Additionally, few studies have used the internationally recognized standard definition of MetS.

Accordingly, we aimed to explore whether MetS could affect postoperative complications and the long-term survival of patients with CRC based on the standard Chinese Diabetes Society (CDS) criteria (10).

## 2 Material and methods

### 2.1 Patients

We retrospectively collected data regarding patients with CRC who had undergone radical surgery at the Second Affiliated Hospital of Wenzhou Medical University from January 2016 to December 2018. The inclusion criteria were as follows: (1) complete medical records in the electronic medical system, (2) age > 18 years, (3) having undergone radical CRC resection, and (4) postoperative confirmation of the CRC diagnosis. The exclusion criteria were as follows: (1) having received preoperative antitumor therapy, including chemoradiotherapy; (2) having a history of other tumors; and (3) having undergone palliative CRC surgery.

### 2.2 Data collection

We collected the following baseline data of the patients with CRC from the electronic medical system: (1) clinicopathological features, including sex, age, preoperative perforation, preoperative obstruction, preoperative bleeding, tumor location, surgical history, Charlson comorbidity index, preventive colostomy, tumor-node-metastasis (TNM) stage (8th edition of the American Joint Committee on Cancer) (11), tumor differentiation, and combined resection as well as (2) postoperative complications (Clavien-Dindo classification grade) (12), including gastrointestinal disorders, wound infection, bleeding, intra-abdominal abscess, anastomotic leakage, intestinal obstruction, and urinary retention.

### 2.3 Follow-up

After discharge, follow-up telephone interviews were conducted at 3-month intervals until the patient’s death or until the follow-up deadline in January 2021. Physical examination, laboratory examination, and computed tomography were performed during the follow-up visit. OS was determined from the date of CRC resection. During the follow-up period, the survival time, tumor recurrence time, and mortality causes were recorded. We excluded patients with CRC with a follow-up period of < 2 years.

### 2.4 Definition of MetS

We used the CDS criteria to define the MetS as follows: (1) systolic blood pressure ≥ 140 mmHg, diastolic blood pressure ≥ 90 mmHg, or specific treatment for previously diagnosed hypertension; (2) fasting blood glucose ≥ 6.1 mmol/L or blood glucose ≥ 7.8 mmol/L after 2 h in the oral glucose tolerance test, or specific treatment for type 2 diabetes mellitus; (3) triglyceride (TG) ≥1.7 mmol/L; (4) body mass index (BMI) ≥ 25 kg/m^2^; and (5) high-density lipoprotein cholesterol (HDL-C) < 0.9 mmol/L for males or < 1.0 mmol/L for females (10). Patients who met three or more of these criteria were diagnosed with MetS.

### 2.5 Statistical analysis

Propensity scores were generated using a logistic regression model based on age, Charlson comorbidity index, preventive colostomy, TNM stage, and tumor differentiation. Propensity score matching (PSM) was performed at a 1:2 ratio with a caliper size of 0.05. Normally and non-normally distributed continuous variables are presented as the mean with standard deviation (SD) and median with interquartile range, respectively. Student’s t-test and Mann–Whitney U test were used for between-group comparisons of normally and non-normally distributed variables, respectively. Categorical variables were compared using the chi-squared test or Fisher’s exact test. Conditional logistic regression analysis was used to evaluate the relationship between various clinicopathological features and postoperative complications. OS was calculated from the date of CRC resection to the date of death or last available follow-up. OS was estimated using Kaplan-Meier method while survival curves were compared using the log-rank test. Univariate and multivariate analyses of various clinicopathological factors were performed using the Cox-Mantel log-rank test, with OS as the dependent factor. Statistical analyses were performed using SPSS Statistics software for Windows (version 22.0; IBM Corp., Armonk, NY, USA).

## 3 Results

### 3.1 Patient characteristics

**Figure 1:**
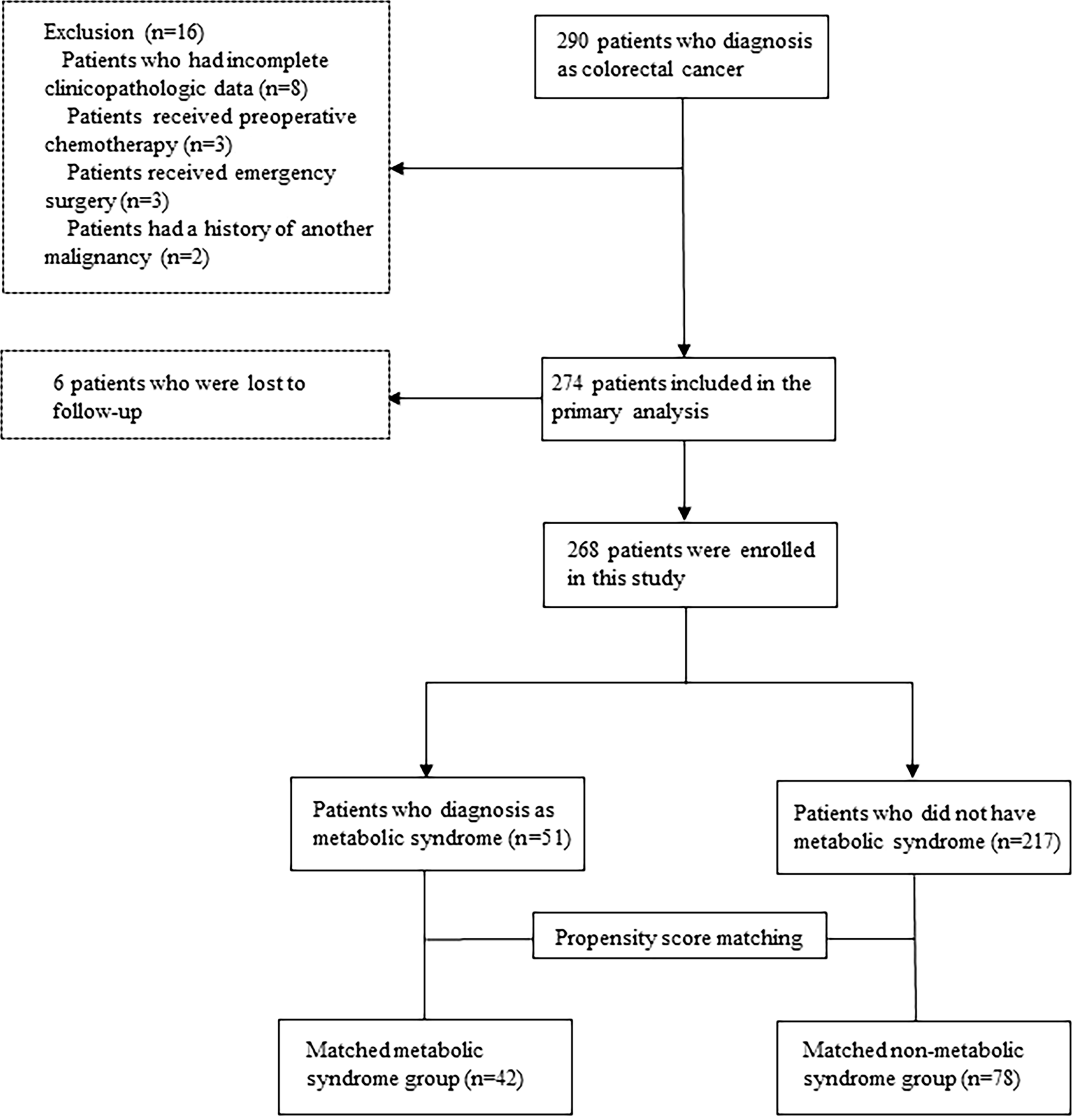
Flow chart of the study procedure.

The median follow-up period was 40 months. Among 290 initially recruited patients, we excluded 22 patients based on the reasons shown in **Figure 1**; accordingly, 268 patients were enrolled. As shown in **Table 1**, there were significant between-group differences in the incidence of hypertension and diabetes, BMI, and TG and HDL-C levels. **Table 2** shows the baseline characteristics of both groups. Based on the CDS diagnostic criteria, 19.03% (51/268) of the patients were diagnosed with MetS. Compared with patients without MetS, patients with MetS were older, more likely to have comorbidities, and underwent preventive colostomy. There were no significant between-group differences in the other characteristics, including sex, incidence of intestinal perforation/obstruction, TNM stage, and tumor differentiation. After PSM, the total cohort comprised 120 patients, including 42 and 78 patients in the MetS and non-MetS groups, respectively. As shown in **Table 2**, both groups were well matched after PSM, with no significant between-group differences in clinicopathological features.

**Table 1:** Factors associated with metabolic syndrome.

Factors	Total (n=268)	MetS (n=51)	Non-MetS (n=217)	P
**Hypertension**				<0.001*
No	176 (65.67%)	10 (19.61%)	166 (76.50%)	
Yes	92 (34.33%)	41 (80.39%)	51 (23.50%)	
**Diabetes mellitus**				<0.001*
No	203 (75.75%)	18 (35.29%)	185 (85.25%)	
Yes	65 (24.25%)	33 (64.71%)	32 (14.75%)	
**BMI (Kg/m^2^, Mean ± SD)**	22.43 ± 3.23)	24.63 ± 2.83	21.92 ± 3.10	<0.001*
**BMI**				<0.001*
Low	48 (17.91%)	26 (50.98%)	195 (89.86%)	
High	220 (82.09%)	25 (49.02%)	22 (10.14%)	
**TG(mmol/L, Mean ± SD)**	1.44 ± 0.97	2.19 ± 1.13	1.26 ± 0.84	<0.001*
**TG**				<0.001*
Low	67 (25.00%)	14 (27.45%)	164 (75.58%)	
High	201 (75.00%)	37 (72.55%)	53 (24.42%)	
**HDL-C (mmol/L, Mean ± SD)**	1.07 ± 0.30	0.89 ± 0.23	1.12 ± 0.30	<0.001*
**HDL-C**				<0.001*
Low	162 (60.45%)	10 (19.61%)	152 (70.05%)	
High	106 (39.55%)	41 (80.39%)	65 (29.95%)	

Data are expressed as number of patients unless indicated otherwise.

MetS, metabolic syndrome; BMI, body mass index; TG, triglyceride; HDL-C, high-density lipoprotein cholesterol.

*Statistically significant (P < 0.05).

**Table 2:** Patient baseline characteristics.

Factors	Unmatched comparison			Matched comparison		
	MetS (n=51)	Non-MetS (n=217)	P	MetS (n=42)	Non-MetS (n=78)	P
**Gender**			0.850			0.984
Female	20 (39.22%)	82 (37.79%)		15 (35.71%)	28 (35.90%)	
Male	31 (60.78%)	135 (62.21%)		27 (64.29%)	50 (64.10%)	
**Age**			0.018*			0.791
≥65	35 (68.63%)	109 (50.23%)		29 (69.05%)	52 (66.67%)	
<65	16 (31.37%)	108 (49.77%)		13 (30.95%)	26 (33.33%)	
**Preoperative perforation**			1.000			1.000
Yes	2 (3.92%)	6 (2.76%)		2 (4.76%)	3 (3.85%)	
No	49 (96.08%)	211 (97.24%)		40 (95.24%)	75 (96.15%)	
**Preoperative obstruction**			0.320			0.368
Yes	5 (9.80%)	33 (15.21%)		4 (9.52%)	12 (15.38%)	
No	46 (90.20%)	184 (84.79%)		38 (90.48%)	66 (84.62%)	
**Preoperative bleeding**			0.437			0.419
Yes	28 (54.90%)	132 (60.83%)		21 (50.00%)	45 (57.69%)	
No	23 (45.10%)	85 (39.17%)		21 (50.00%)	33 (42.31%)	
**Tumor location**			0.179			0.102
Right colon	11 (21.57%)	39 (17.97%)		9 (21.43%)	19 (24.36%)	
Left colon	19 (37.25%)	58 (26.73%)		18 (42.86%)	19 (24.36%)	
Rectum	21 (41.18%)	120 (55.30%)		15 (35.71%)	40 (51.28%)	
**Tumor size (diameter, cm)**	**4.28 ± 1.69**	**4.45 ± 1.68**	**0.512**	**4.38 ± 1.61**	**4.43 ± 1.50**	**0.870**
**Surgical history**			0.639			0.872
Yes	6 (11.76%)	31 (14.29%)		6 (14.29%)	12 (15.38%)	
No	45 (88.24%)	186 (85.71%)		36 (85.71%)	66 (84.68%)	
**Charlson comorbidity Index**			<0.001*			0.779
≥1	35 (68.63%)	70 (32.26%)		28 (66.67%)	50 (64.10%)	
0	16 (31.37%)	147 (67.74%)		14 (33.33%)	28 (35.90%)	
**Preventive colostomy**			0.032*			1.000
Yes	9 (17.65%)	15 (6.91%)		1 (2.38%)	1 (1.28%)	
No	42 (82.35%)	202 (93.09%)		41 (97.62%)	77 (98.72%)	
**TNM stage**			0.474			0.945
I	13 (25.49%)	39 (17.97%)		7 (16.67%)	14 (17.95%)	
II	17 (33.33%)	80 (36.87%)		17 (40.48%)	33 (42.31%)	
III	21 (41.18%)	98 (45.16%)		18 (42.86%)	31 (39.74%)	
**Differentiation**			0.685			0.885
High/Middle	43 (84.31%)	188 (86.64%)		37 (88.10%)	68 (87.18%)	
Low	8 (15.69%)	29 (13.36%)		5 (11.90%)	10 (12.82%)	
**Combined resection**			0.944			1.000
Yes	3 (5.88%)	16 (7.37%)		3 (7.14%)	7 (8.97%)	
No	48 (94.12%)	201 (92.63%)		39 (92.86%)	71 (91.03%)	

MetS, metabolic syndrome; TNM, tumor–node–metastasis.

*Statistically significant (P<0.05, two sides).

### 3.2 The relationship between MetS and postoperative complications

In the unmatched cohort, 94 of 268 (35.07%) patients presented postoperative complications (**Table 3**). The MetS group had a significantly higher incidence of postoperative complications than the non-MetS group (49.02% vs. 31.80%, P = 0.002). Specifically, the MetS group had a significantly higher incidence of severe complications and surgical complications than the non-MetS group (33.33% vs. 18.43%, P = 0.019; 33.33% vs. 16.59%, P = 0.007, respectively). However, after PSM, the MetS group had a non-significantly higher postoperative complication rate.

**Table 3:** Patients’ postoperative complications.

Factors	Unmatched comparison			Matched comparison		
	MetS (n=51)	Non-MetS (n=217)	P	MetS (n=42)	Non-MetS (n=78)	P
Total complications	25 (49.02%)	69 (31.80%)	0.020*	18 (42.86%)	33 (42.31%)	0.954
Clavien-Dindo grade						
Grade I	8 (15.69%)	29 (13.36%)	0.665	6 (14.29%)	14 (17.95%)	0.608
Grade II	13 (25.49%)	32 (14.75%)	0.065	9 (21.43%)	17 (21.79%)	0.963
Grade III	3 (5.88%)	8 (3.69%)	0.750	3 (7.14%)	2 (2.56%)	0.342
Grade IV	1 (1.96%)	0 (0%)	0.190	0 (0%)	0 (0%)	–
Severe complications^a^	17 (33.33%)	40 (18.43%)	0.019*	12 (28.57%)	19 (24.34%)	0.615
Detail of complications						
**Surgical complications**	17 (33.33%)	36 (16.59%)	0.007*	11 (26.19%)	16 (20.51%)	0.676
Gastrointestinal disorders	4 (7.84%)	8 (3.69%)		4 (9.52%)	4 (5.13%)	
Wound infection	4 (7.84%)	7 (3.23%)		2 (4.76%)	3 (3.85%)	
Bleeding	1 (1.96%)	5 (2.30%)		1 (2.38%)	1 (1.28%)	
Intra-abdominal abscess	5 (9.80%)	3 (1.38%)		1 (2.38%)	3 (3.85%)	
Anastomotic leakage	2 (3.92%)	7 (3.23%)		2 (4.76%)	2 (2.56%)	
Intestinal obstruction	1 (1.96%)	3 (1.38%)		0 (0%)	1 (1.28%)	
Urinary retention	2 (3.92%)	3 (1.38%)		1 (2.38%)	2 (2.56%)	
**Medical complications**	13 (25.49%)	38 (17.51%)	0.191	10 (23.81%)	21 (26.92%)	0.710
Pulmonary infection	4 (7.84%)	14 (6.45%)		4 (9.52%)	9 (11.54%)	
Cardiac complications	1 (1.96%)	0 (0%)		1 (2.38%)	0 (0%)	
Venous thrombosis	1 (1.96%)	3 (1.38%)		1 (2.38%)	1 (1.28%)	
Urinary infection	0 (0%)	2 (0.92%)		1 (2.38%)	1 (1.28%)	
Fever	1 (1.96%)	7 (3.23%)		1 (2.38%)	3 (3.85%)	
Transfusion^b^	8 (15.69%)	22 (10.14%)		7 (16.67%)	12 (15.38%)	
Stroke	2 (3.92%)	0 (0%)		0 (0%)	0 (0%)	
30-day mortality	2 (3.92%)	1 (0.46%)	0.094	2 (4.76%)	0 (0%)	0.121
Postoperative hospital stays [days, median (IQR)]	17.00 (7.00)	15.00 (5.50)	0.081	16.00 (7.25)	16.00 (6.25)	0.945
Hospitalization costs [￥, median (IQR)]	50872.28 (21798.74)	53078.04 (21029.92)	0.915	50914.14 (24,787.19)	54772.17 (25061.78)	0.878

a Clavien-Dindo grade≥II.

b Including albumin and/or erythrocyte.

MetS, metabolic syndrome; TNM, tumor–node–metastasis; IQR, interquartile range.

*Statistically significant (P<0.05, two sides).

### 3.2 The relationship between MetS and OS

**Figure 2:**
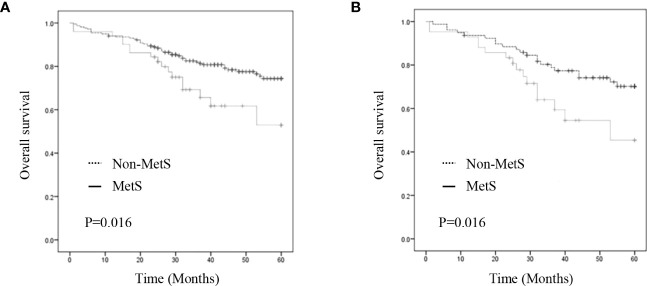
Kaplan–Meier curve for overall survival before and after matching. **(A)** Kaplan–Meier curve for overall survival of unmatched group **(B)** Kaplan–Meier curve for overall survival of matched group.

As shown in **Figure 2**, the MetS group had a shorter OS than the non-MetS group (P = 0.016 and P = 0.027, respectively). After matching, univariate analysis revealed that intestinal obstruction (HR 2.743, P = 0.009), high TNM stage (HR 2.991, P = 0.001), low differentiation (HR 2.207, P = 0.048), and MetS (HR 2.075, P = 0.031) were associated with worse OS. In multivariate analysis, intestinal obstruction (HR 2.761, 95% CI 1.280-5.955, P = 0.010), TNM stage (HR 2.422, 95% CI 1.351-4.342, P = 0.003), and MetS (HR 1.997, 95% CI 1.026-3.888, P = 0.042) were independent risk factors for OS (**Table 4**). On the other hand, as for the MetS parameters, we found hypertension (HR 2.256, P=0.001), diabetes mellitus (HR 1.713, P=0.047) and High HDL-C (HR 2.262, P=0.001) was associated with worse OS, while only hypertension (HR 1.655, P=0.025), diabetes mellitus (HR 1.672, P=0.035)were risk factors for OS.(**Table 5**).

**Table 4:** Univariate and multivariate Cox regression analysis of factors associated with overall survival.

Factors	Unmatched comparison				Matched comparison			
	Univariate analysis		Multivariate analysis		Univariate analysis		Multivariate analysis	
	HR (95% CI)	P	HR (95% CI)	P	HR (95% CI)	P	HR (95% CI)	P
Gender								
Female	Ref				Ref			
Male	0.900 (0.543-1.491)	0.682			1.090 (0.555-2.142)	0.802		
Age								
≥65	Ref		Ref		Ref			
<65	2.044 (1.203-3.476)	0.008*	2.173(1.244-3.796)	0.006*	1.651 (0.779-3.501)	0.191		
Preoperative perforation								
No	Ref				Ref			
Yes	0.878 (0.214-3.599)	0.856			0.470 (0.064-3.432)	0.456		
Preoperative obstruction								
No	Ref				Ref		Ref	
Yes	2.524 (1.427-4.464)	0.001*	1.877(1.051-3.353)	0.033*	2.743 (1.283-5.807)	0.009*	2.761 (1.280-5.955)	0.010*
Preoperative bleeding								
No	Ref				Ref			
Yes	1.058 (0.793-1.411)	0.702			0.750 (0.393-1.430)	0.382		
Tumor location								
Rectum	Ref				Ref			
Right colon	1.380 (0.712-2.675)	0.340			0.978 (0.422-2.260)	0.958		
Left colon	1.682 (0.964-2.933)	0.067			1.045 (0.499-2.190)	0.906		
Surgical history								
No	Ref				Ref			
Yes	1.138 (0.561-2.307)	0.720			0.955 (0.371-2.456)	0.924		
Charlson Index								
0	Ref				Ref			
≥1	1.839 (1.120-3.018)	0.016*	1.855 (1.100-3.129)	0.021*	1.552 (0.765-3.190)	0.224		
**TNM stage**	2.291(1.530-3.431)	0.001*	2.289 (1.491-3.512)	<0.001	2.991(1.454-4.269)	0.001*	2.422 (1.351-4.342)	0.003*
Differentiation								
High/Middle	Ref				Ref		Ref	
Low	2.563 (1.453-4.522)	0.001*	2.314 (1.258-4.527)	0.007*	2.207(1.008-4.833)	0.048*	1.116 (0.478-2.604)	0.800
Combined resection								
No	Ref				Ref			
Yes	0.997 (0.362-2.745)	0.995			1.066 (0.327-3.475)	0.915		
MetS								
No	Ref		Ref		Ref		Ref	
Yes	1.963 (1.120-3.439)	0.018*	1.459 (0.799-2.666)	0.050	2.075(1.068-4.032)	0.031*	1.997 (1.026-3.888)	0.042*

MetS, metabolic syndrome; TNM, tumor–node–metastasis.

*Statistically significant (P<0.05, two sides).

**Table 5:** Univariate and multivariate Cox regression analysis of factors associated with overall survival.

Factor	Unmatched		Matched	
	HR (95% CI)	P	HR (95% CI)	P
Hypertension				
No	Ref		Ref	
Yes	2.256 (1.374-3.706)	0.001*	1.655 (1.066-2.569)	0.025*
Diabetes mellitus				
No	Ref		Ref	
Yes	1.713 (1.006-2.918)	0.047*	1.672 (1.038-2.692)	0.035*
High BMI				
No	Ref		Ref	
Yes	0.814 (0.414-1.601)	0.551	1.030 (0.617-1.720)	0.909
High TG				
No	Ref		Ref	
Yes	1.310 (0.757-2.264)	0.334	1.168 (0.726-1.880)	0.522
High HDL-C				
No	Ref		Ref	
Yes	2.262 (1.374-3.722)	0.001*	1.151 (0.747-1.774)	0.524

MetS, metabolic syndrome; TNM, tumor–node–metastasis.

*Statistically significant (P<0.05, two sides).

## 4 Discussion

Previous studies have used numerous different indicators and criteria to describe MetS. Specifically, some studies have used various indicators, including BMI, as a discriminatory component for MetS to evaluate the body’s metabolic status (13, 14). However, BMI, which is an obesity indicator, is inadequate as a sole indicator of metabolic disorder. Milano et al. reported that MetS, but not hypertension or obesity, was significantly associated with CRC (15). There are several definitions of MetS, including the National Cholesterol Education Program–Adult Treatment Panel III criteria (16), International Diabetes Federation criteria (17), CDS criteria (10), and the Joint Interim Statement criteria (18). Numerous studies have used different definitions of MetS as well as modifications of the listed MetS criteria (8, 9), which might affect the reliability of the results. We used the standard CDS criteria since they are more consistent with the characteristics of the Chinese population. In our study, the prevalence of CRC in the patients with MetS was 19.03% (51/268).

To our knowledge, this is the first study to comprehensively analyze the impact of MetS on the short- and long-term prognoses of patients with CRC. Consistent with previous findings, we found that the patients with MetS were older and had more preoperative comorbidities (8); further, MetS could adversely affect OS. However, there was no direct evidence of an effect of MetS on postoperative complications.

In the unmatched cohort, MetS was associated with postoperative and surgical complications, which is consistent with the report by Zhou et al. (8, 9). However, after PSM, we found that MetS was not associated with postoperative complications, which is consistent with the report by Goulart et al. (19, 20). Therefore, it remains unclear whether MetS affects postoperative complications in patients with CRC, which could be attributed to differences in the diagnostic criteria of MetS across studies or confounding factors. However, this is the first related study to use the PSM method, which reduced the influence of other confounding factors on the results.

Few studies have explored the relationship between MetS and long-term survival in patients with CRC. In our study, the MetS group had a significantly shorter OS than the non-MetS group; moreover, MetS was an independent risk factor for postoperative long-term survival. MetS could affect OS through several mechanisms. First, MetS-induced insulin resistance affects the normal metabolism of adipocytes, which increases the levels of pro-inflammatory substances and decreases the levels of adiponectin (a protective adipokine), and thus resulting in a long-term chronic inflammatory state (21). Second, dyslipidemia, abnormal blood glucose, and hypertension are related to damage to the microvascular circulation, which results in multiple organ dysfunction (22). Third, abdominal obesity adversely affects surgical exposure and anatomy, which may cause incomplete lymph node dissection during operation (23). These aforementioned pathophysiological mechanisms may negatively affect the OS of patients with CRC.

We found that independent risk factors, including intestinal obstruction and TNM stage, were difficult to alter through preoperative interventions. As the only independent risk factor, MetS significantly influences long-term postoperative survival. Recent studies have demonstrated that short-term preoperative lifestyle interventions could effectively reduce or even reverse MetS (24). Therefore, future studies should focus on whether preoperative interventions can improve the prognosis of patients with CRC by reversing MetS.

This study has several limitations. First, the sample size was small, which might have affected the reliability of our results. Second, this was a single-center retrospective study that only enrolled Chinese patients, which might have led to some biases. Further, A large portion of the patients were diagnosed before admission and were prescribed the corresponding therapeutic drugs, the diagnosis of Mets was based on both of medical history and hospitalization tests. We overlook the impact of medication history on patients’ metabolic status. The last and the most important, since a part of patients chose to go to better medical centers for further treatment and another part didn’t receive regular postoperative review and treatment, we could not obtain the exactly recurrence time. The analysis of DFS and RFS were thus missed. Thus, future large-scale, multicenter, prospective, and interventional studies are warranted.

In conclusion, MetS can affect the long-term survival of patients with CRC. Therefore, preoperative lifestyle interventions for reducing or reversing MetS are strongly recommended.

## Data availability statement

The raw data supporting the conclusions of this article will be made available by the authors, without undue reservation.

## Author contributions

CZ, LL and XS are the main authors of manuscript and have made substantial contributions to the conception and design of study. CM, WC, JZ, HY, JC, TY, YY have been involved in collection and analysis of the data, and CZ wrote this manuscript. CM, LL and XS gave final approval and revised of the manuscript. All authors contributed to the article and approved the submitted version.
